# Surgical Treatment of Bone Sarcoma

**DOI:** 10.3390/cancers14112694

**Published:** 2022-05-29

**Authors:** Felix Bläsius, Heide Delbrück, Frank Hildebrand, Ulf Krister Hofmann

**Affiliations:** 1 Department of Orthopaedic, Trauma and Reconstructive Surgery, RWTH Aachen University Hospital, Pauwelsstraße 30, 52074 Aachen, Germany; fblaesius@ukaachen.de (F.B.); hdelbrueck@ukaachen.de (H.D.); fhildebrand@ukaachen.de (F.H.); 2 Centre for Integrated Oncology Aachen Bonn Köln Düsseldorf (CIO), 52074 Aachen, Germany

**Keywords:** osteosarcoma, Ewing sarcoma, chondrosarcoma, tumor surgery, rotationplasty, megaprosthesis, tumor resection, bone

## Abstract

**Simple Summary:**

Even today, a malignant bone tumor is still a threatening condition for the patient. Such tumors are difficult to treat and they require an interdisciplinary approach to ensure the best possible outcome. This review article provides an overview of the three dominating bone sarcoma entities: osteosarcoma, chondrosarcoma, and Ewing sarcoma. Their prognoses and main pillars of treatment (chemotherapy, radiotherapy, surgery) are laid out with a special focus on the surgical management of this condition. Five cases are described to illustrate different surgical strategies such as biological reconstruction and implantation of a megaprosthesis. Finally, an outline of future developments in the field of tumor surgery is presented with a special focus on technical innovations to help improve surgical outcome and implant survival.

**Abstract:**

Bone sarcomas are rare primary malignant mesenchymal bone tumors. The three main entities are osteosarcoma, chondrosarcoma, and Ewing sarcoma. While prognosis has improved for affected patients over the past decades, bone sarcomas are still critical conditions that require an interdisciplinary diagnostic and therapeutic approach. While radiotherapy plays a role especially in Ewing sarcoma and chemotherapy in Ewing sarcoma and osteosarcoma, surgery remains the main pillar of treatment in all three entities. After complete tumor resection, the created bone defects need to be reconstructed. Possible strategies are implantation of allografts or autografts including vascularized bone grafts (e.g., of the fibula). Around the knee joint, rotationplasty can be performed or, as an alternative, the implantation of (expandable) megaprostheses can be performed. Challenges still associated with the implantation of foreign materials are aseptic loosening and infection. Future improvements may come with advances in 3D printing of individualized resection blades/implants, thus also securing safe tumor resection margins while at the same time shortening the required surgical time. Faster osseointegration and lower infection rates may possibly be achieved through more elaborate implant surface structures.

## 1. Introduction

Bone sarcomas are primary malignant bone tumors that are characterized by sarcomatous tumor cells associated with the production of osteoid matrix or bone. They can be further classified according to their cytologic features into the three dominating entities: osteosarcomas (up to 40%), chondrosarcomas (20–27%), and Ewing sarcomas (up to 15%) [1,2] (Table 1). Other and unspecified bone tumors comprise another approximately 25%.

Bone sarcomas are rare diseases with annual incidences of around 800 cases in Germany and 3600 cases in the U.S., thus accounting for approximately 0.2% of all neoplasms registered in the EUROCARE database [5]. With respect to their main age of occurrence, relevant differences can be observed: Osteosarcomas are typically diagnosed in the time of adolescence (10–14 years) or they are found in the elderly population (older than 65 years). Chondrosarcomas, in contrast, are most frequently diagnosed at an age of 40–60 years. The third entity, Ewing sarcoma, is a malignancy that is found in the second decade of life with a peak around 15 years of age [6]. Bone sarcoma cells are cells of the mesenchymal lineage. Osteosarcomas are characterized by the expression of osteoblastic markers (e.g., phosphatase and osteocalcin), whereas chondrosarcomas can be related to a chondrocytic genesis (e.g., type II collagen or aggrecan) by their surface markers. Ewing sarcomas have a special role as their origin is not quite clear. Ewing sarcomas are characterized by fusion proteins (usually the *EWS* gene and *ETS* family members). In the past 15 years, the characterization of Ewing sarcoma has made significant progress, so that currently three main categories are defined: *EWSR1*-non-*ETS* fusions, *CIC*-related sarcoma, and sarcoma with *BCOR* gene alterations. The evolution of Ewing sarcoma entities continues to progress and will result in future changes regarding classification [3,7]. Popular stem cell theories represent a refinement of these etiological origin theories, according to which subpopulations of bone sarcoma cells exhibit stem cell-like features (e.g., self-renewal and differentiation) that can also be found in embryonic stem cells [8].

The grading of malignant bone tumors is based on the histopathological findings. Two equally well-known grading systems are available: FNCLCC (French Féderation Nationale des Centres de Lutte Contre le Cancer) grading and UICC (Union for International Cancer Control) grading. While initially differing in the number of total possible grades (FNCLCC 1–3, UICC 1–4), this discrepancy was resolved in the latest WHO classification, with only three grades being now in use (Table 2) [3,9]. In addition, the TNM classification is used to describe local tumor extent (T), presence of pathological lymph nodes (N), and existence of metastases (M) [8]. The number of available grading systems with quite different criteria shows that grading continues to be a challenge. The revised version of the WHO Classification of Tumours (5th Edition) takes this into consideration and integrates the discussion with respect to these differences into its latest release. In addition to the established histopathological methods, next-generation sequencing technologies are becoming increasingly valuable in further specifying the tumor entities and thus contributing to classification, diagnostics, and targeted therapy, although specific therapies are, to date, only available for a few mutations [10].

Osteosarcoma is the most frequent type of bone sarcoma (approx. 320 cases/year in Germany and approx. 1550 cases/year in the United States [5]). When detected early with a low disease burden (non-metastatic), local surgical R0 resection accompanied by early initiation of a modern chemotherapy regimen can achieve a long-term survival in two-thirds of patients (Salzer-Kuntschik regression grade >3: 5-year survival rate 50%; ≤3: 5-year survival rate 80%) [12]. In Europe, the choice of the chemotherapeutic treatment is made according to evidence-based results from the EURAMOS-1, EURO-B.O.S.S., or COSS-96 studies. Therapeutic regimens include the combination of doxorubicin, cisplatin, ifosfamide, and methotrexate in the framework of neoadjuvant and adjuvant chemotherapy [13,14,15,16]. Chondrosarcomas represent the second most common bone sarcoma entity (approximately 160 cases/year in Germany). Low-grade lesions are treated by local curettage, and high-grade lesions are addressed by wide resection. Radiation and chemotherapy currently only have a role in palliative settings due to the relative radio- and chemotherapy resistance of this entity. Treatment of Ewing sarcomas includes neoadjuvant chemotherapy followed by surgery and/or local radiation therapy. Subsequent adjuvant chemotherapy is also administered as part of clinical studies (e.g., Euro-E.W.I.N.G. 99, EWING-2008 study) [17,18]. The neoadjuvant chemotherapy includes six cycles of VIDE (vincristine, ifosfamide, doxorubicin, etoposide) followed by eight cycles of VAC (vincristine, actinomycin D, cyclophosphamide) or VAI (vincristine, actinomycin D, ifosfamide) after local therapy has been performed. The use of treosulfan/melphalan was investigated for high-risk patients in the EWING-2008 study [19], which found no advantages in adults but suggested a possible benefit for a subgroup of patients younger than 14 years [20].

In tumor surgery, four different extensions of surgical resection have been established: intralesional, marginal, wide, and radical resection [21]. The primary goal in surgery of bone sarcomas is complete tumor resection (R0). The Musculoskeletal Tumor Society, therefore, recommends wide local resection or amputation in these cases [22,23]. With modern treatment strategies, limb preservation is often, but not always, possible. Randomized, controlled trials comparing the use of a limb-preserving approach with amputation are absent. However, retrospective studies could not demonstrate a difference between both strategies in terms of total survival [24].

Except for Ewing sarcomas, bone sarcomas show a relative radio-resistance [17,25,26,27]. One possible reason for this observation could be stem cell-like tumor cell populations, which exhibit a high regenerative potency [25,28]. Therefore, surgical therapy and (neo-)adjuvant chemotherapy represent the two main pillars of bone sarcoma treatment. Meticulous preoperative evaluation (confirmation of histologic diagnosis, assessment of disease extent, definition of treatment goals, feasibility of limb preservation) is critical for targeted surgical therapy. Preoperative imaging includes local radiographs, staging CT (80% of bone sarcoma metastases are found in the lungs [29]), and local MR imaging. In this context, CT of the thorax and whole-body scintigraphy are mandatory for the search for distant metastases. The diagnosis can optionally be extended by abdominal ultrasonography or CT of the abdomen/pelvic region as well as bone marrow histology with subsequent next-generation sequencing (e.g., Ewing sarcoma t(11;22) [30,31,32], see study protocol EURO-E.W.I.N.G. 99 [18]). MR imaging should include native T1w, STIR, and T2w(-TSE/SE) sequences as well as contrast agent-enhanced imaging, as typical enhancements may be observed depending on the tumor entity (e.g., chondrosarcoma: ring or arc enhancement) [33]. In some cases, also a PET-CT or, in younger patients, a PET-MRI may also be justified. As standard tracer fluorodeoxyglucose (FDG) is usually used. Most data on the utility of this modern but also expensive means of diagnostic are available on soft tissue sarcoma, and its role in bone sarcoma still needs to be evaluated [34]. A biopsy of the tumor completes the staging process. Although, in many cases, seemingly trivial, this is a critical step that must be carefully planned [35]. Due to the menacing nature of the condition, it is essential to obtain a representative sample of the tumor. Moreover, the biopsy approach must be chosen in such a way that it will be excised during the definitive surgical procedure. During the procedure, meticulous hemostasis is essential to prevent the spread of malignant cells from the tumor site into surrounding tissues [36]. For these reasons, open biopsy is considered the standard, especially as CT-guided, fine-needle biopsies have a high rate of false-negative results [37]. The histopathological workup includes, first, conventional histology and immunohistochemistry, which is then followed by molecular confirmation by, for example, RNA analysis [7].

Since bone sarcomas are rare diseases, patients are treated at specialized tumor centers and they are generally included in multicenter, controlled studies. Patients treated in high-volume centers have a 15% lower mortality risk compared to patients treated at low-volume centers. Additionally, the likelihood of limb-salvage surgery is higher at specialized centers [38].

In the following three sections, the different entities will be described in more detail and they will be illustrated by clinical cases. The surgical techniques described in the corresponding sections are typical for the entities under which they are mentioned and they are explained in context with the provided cases. They can, however, also be applied in the other bone sarcomas, provided that they are suited for the specific case.

## 2. Osteosarcoma

### 2.1. Prognosis

#### 2.1.1. High-Grade Osteosarcoma in <40-Year-Old Patients

Between April 2005 and June 2011, 2260 patients were registered in the European and American Osteosarcoma Study (EURAMOS)-1, and the following updated results were concluded (Smeland et al., 2019 [39]): The 3-year and 5-year overall survivals were 79% and 71%, respectively. Most adverse factors at diagnosis were pulmonary metastases, non-pulmonary metastases, or an axial skeleton tumor site. The histological subtypes, telangiectatic and unspecified conventional osteosarcoma, were associated with a favorable prognosis compared with a chondroblastic subtype. Poor histological response after neoadjuvant chemotherapy was associated with a worse outcome after surgery.

#### 2.1.2. High-Grade Osteosarcoma in >40-Year-Old Patients

The EUROpean Bone Over 40 Sarcoma Study (EURO-B.O.S.S.) was the first international prospective study for patients 41–65 years old with high-grade bone sarcoma. Its design was derived from the protocols of younger patients. The 5-year overall survival in patients with localized disease was 29% in pelvic tumors, 70% for tumors of the extremities, and 73% for craniofacial locations [13].

#### 2.1.3. Low-Grade Osteosarcoma/Parosteal Osteosarcoma

In a monocentric study analyzing 195 patients with parosteal osteosarcoma [40], 5-year (65% versus 96%) and 10-year survivals (60% versus 96%) were lower for those patients who suffered from dedifferentiated parosteal osteosarcoma when compared to parosteal osteosarcoma without dedifferentiation. Wide surgical margins played a relevant role for the length of both disease-free and overall survival. Interestingly, tumor size, age at presentation, and medullary involvement did not influence patient survival. The strongest negative effect on clinical outcome was observed for the degree of dedifferentiation.

### 2.2. Therapy

More than 80% of patients who undergo surgical therapy alone develop metastases despite local tumor control [41]. This is currently attributed to occult metastases at the time of diagnosis. Therefore, adjuvant chemotherapy is mandatory. Attempting a neoadjuvant chemotherapy can provide information on whether the tumor is chemotherapy sensitive. This represents an independent prognostic factor, with a positive response being considered prognostically favorable. However, reducing the tumor burden through a neoadjuvant chemotherapy should not tempt changing the initially planned surgical strategy (set after staging). This regularly leads to undertreatment and bears a high risk of disease progression due to the lack of an aggressive surgical therapy with a wide resection with safe R0 margins.

The present therapy concept of high-grade osteosarcomas includes about 10 weeks of neoadjuvant chemotherapy based on the results of the EURAMOS-1 study [16] with the cytostatics methotrexate, Adriamycin^®^ (doxorubicin), and cisplatin (MAP) followed by surgery. After the operation, chemotherapy with the abovementioned cytostatics is continued. The postsurgical treatment consists of 12 cycles (a total of 2 cycles of cisplatin/doxorubicin, 2 cycles of doxorubicin, and 8 cycles of methotrexate) and lasts around 6 to 7 months, including the breaks in therapy.

Low-grade osteosarcomas, which are mainly parosteal osteosarcomas, are only treated locally. Radiation therapy presently plays no role in the treatment of osteosarcoma [42].

#### 2.2.1. Surgical Therapy

A main pillar of the treatment of osteosarcoma is surgical therapy with safe tumor-free resection margins. Historically, this goal was usually achieved through radical resection, which, in most cases, meant amputation. With the introduction of systemic therapies for osteosarcoma in the 1970s, limb-sparing procedures emerged, which are now applied in the vast majority of patients. Limb-sparing surgery also requires complete tumor resection, which is then followed by skeletal and/or soft-tissue reconstruction [43].

#### 2.2.2. Limb-Sparing Surgery (LSS) Versus Amputation

From a total of 2442 patients, registered in the National Cancer Database (NCDB) from 2004 through 2015, 1855 underwent limb salvage and 587 underwent amputation [44].

Factors that were associated with a higher rate of amputation in the case of osteosarcoma were large tumor size, advanced stage, advanced age and greater comorbidities, and lower income. Limb salvage, in contrast, was found related with a significant survival benefit, even when controlling for significant confounders and differences between both cohorts. This observation is supported by a meta-analysis from 2016 [45].

#### 2.2.3. Rotationplasty

**Figure 1:**
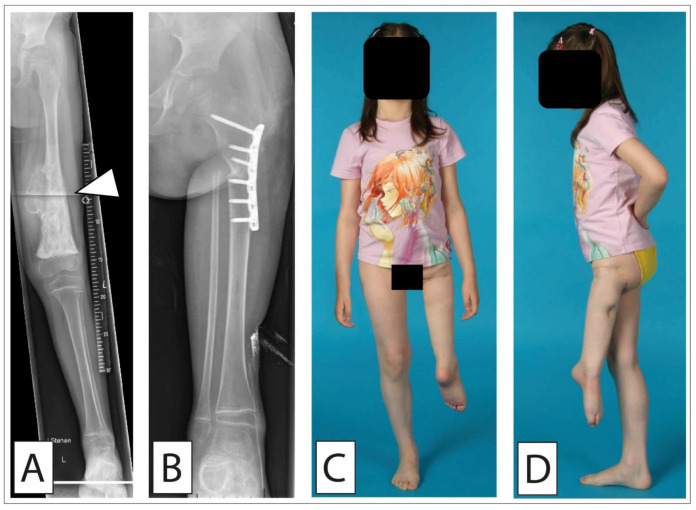
Osteosarcoma of the distal femur treated with rotationplasty. A 5-year-old girl presenting with pain in the left thigh. (**A**) The plain a-p radiograph showed an osseous tumor (white arrow) of the distal left femur highly suggestive for osteosarcoma. Histopathologic analyses after open biopsy confirmed high-grade osteosarcoma. In MR imaging, it could be determined that it was located near vessels and nerves, respecting the epiphyseal plate. Due to the young age and the suitable location, we performed rotationplasty according to the technique of van Nes. Different techniques of rotationplasty have been proposed depending on the location of the tumor in the femur [47]. (**B**) Antero-posterior radiograph showing the osteosynthesis by means of an LCP plate in the subtrochanteric region. Clinical frontal (**C**) and lateral (**D**) image after wound healing.

Especially in younger patients, as an alternative to amputation, rotationplasty is performed (Figure 1). In this procedure, after segment resection of the affected bone, the ankle is transposed in a 180° rotated form to functionally turn into a knee joint with the foot forming the proximal lower leg amputation stump. A study by Gotta et al. [46] examined 60 patients who had undergone rotationplasty due to bone cancer. In a median follow-up of 22 years (range 10–47 years), patients reported a high level of activity with a median Musculoskeletal Tumor Society score of 24 and a mean Tegner activity level scale of 4. Health-Related Quality of Life scores were comparable to the general German population. Concerns of psychological problems due to the unusual appearance of the rotated foot were not confirmed. Since, in the case of a successful procedure, the biologic situation created can support the body for decades, these data support the use of rotationplasty especially in young children even in times of expandable prostheses [46].

#### 2.2.4. Expandable Prostheses

**Figure 2:**
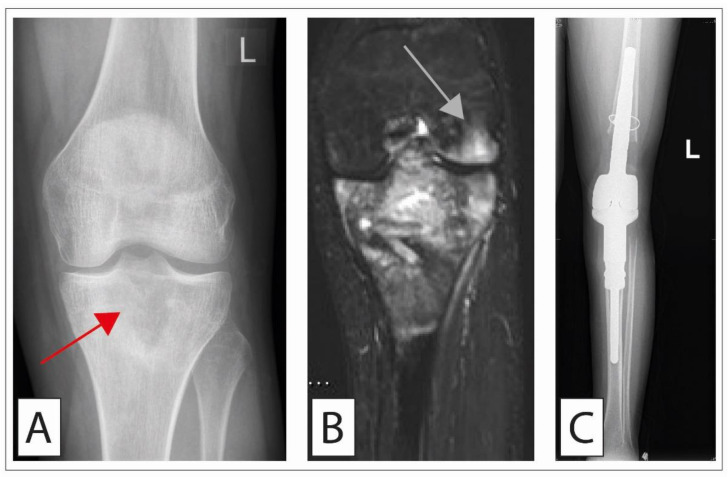
Osteosarcoma treated with extraarticular knee resection and implantation of a megaprosthesis. A 20-year-old patient presenting with pain in the left knee and a progressive extension deficit. (**A**) Conventional radiographs showing osteolysis in the proximal tibia (red arrow). (**B**) MR imaging (T2-tirm coronal) was performed, suggesting that the corresponding medial femoral condyle might also be affected (gray arrow). Intraarticular contamination was, therefore, assumed. Biopsy sampling was performed, showing a G3 osteosarcoma (staging: T1, Nx, M0, IIA). The patient first received neoadjuvant therapy according to the EURAMOS protocol. Thereafter, we performed extraarticular tumor resection [53,54] and implanted a megaprosthesis (Implantcast, Femur RS/KRI and proximal Tibia MUTARS) (**C**). Although parts of the extensor mechanism can be preserved in this procedure, patients postoperatively do complain about problems with sufficient extension. Advanced soft-tissue management was required, with soft-tissue coverage over the proximal tibia being achieved by means of a medial gastrocnemius muscle flap and a medial soleus muscle flap. Histopathological examination showed complete resection of the tumor (R0) and a regression grade of 3 (according to Salzer-Kuntschik), for which reason adjuvant chemotherapy was continued according to the EURAMOS-1 protocol (“good response”).

The alternative for rotationplasty in children with malignant bone sarcoma around the knee is the implantation of an expandable prosthesis that can accommodate for the still expected increase in leg length. Portney et al. [48] published a meta-analysis on the outcome of expandable prostheses for primary bone malignancies in skeletally immature patients including 28 retrospective studies. All studies were of level IV evidence (case series and retrospective studies). In total, 292 cases of individual patient data sets were available from the 634 patients analyzed. Mean age in this subpopulation was 10.1 years at the time of surgery; mean follow-up was 67 months. The main tumor site was the distal femur (216 patients, 74%). Musculoskeletal Tumor Society scores averaged 80.3. Overall mortality was 22%. Several lengthening procedures had to be performed in patients with expandable distal femur replacements (average, 4.4; mean lengthening distance, 43 mm). The overall complication and revision rate was 43%, increasing to 59% in patients with 5 to 10 years of follow-up, and 89% in patients with >10 years of follow-up. Improved outcome results and lower failure rates have, however, been reported with newer generations of expandable prostheses [49]. In this study, the Phenix-Repiphysis system that was used from 1994 to 2008 was compared with the Stanmore JTS non-invasive prosthesis that was used from 2008 to 2016. Functional results were significantly better in the Stanmore group. Patients treated with this device presented with a mean knee flexion of 112 ± 38° and a Musculoskeletal Tumor Society score of 87.6 ± 5.4. With respect to implant survival, the Stanmore group also vastly outperformed the Phenix-Repiphysis system: At the end of follow-up all Stanmore implants were still in place while all Phenix-Repiphysis devices had already been explanted again. Limb length equality was obtained in 79% patients with the Phenix-Repiphysis system and in 84% with the Stanmore implant. Additionally, Windhager et al. [50] state, in their review, satisfactory functional results with an averaged Musculoskeletal Tumor Society score of 78.3 but a high complication rate of 27.3% of infections and 22.4% of mechanical failure. It is this high complication rate that prompts some authors to still argue in favor of rotationplasty [51]. In patients who have almost reached their full body height, the defect resulting from tumor resection is, however, usually treated by the implantation of a regular megaprosthesis (Figure 2). In a case in which the complete knee joint has to be removed in toto, reconstruction of the extensor mechanism can be challenging. Several techniques have been suggested to address this problem, for example, using the gastrocnemius aponeurosis as an augment for the extensor mechanism [52]. One interesting technique is an extraarticular knee resection with preservation of the quadriceps and patella tendon [53,54].

## 3. Ewing Sarcoma

### 3.1. Prognosis

Ewing sarcoma is classified in a heterogeneous group of primitive small round cell tumors. Their origin remains unclear. True Ewing sarcomas are characterized by gene fusions involving at least one member of the FET family. The prognosis for patients with Ewing sarcoma strongly depends on a multitude of factors. Long-term overall survival rates range from less than 30% for patients with extrapulmonary metastases to over 75% for patients without clinically overt metastases at diagnosis. In those patients who present with pulmonary metastases, the 2- to 10-year event-free survival lies around 30–36% within the European Intergroup Cooperative Ewing and Paediatric Oncology Group studies [17]. Multivariable analysis of the European EURO-E.W.I.N.G.-99 trial (EUROpean Ewing tumour Working Initiative of National Groups-Ewing Tumour Studies 1999), on patients with primary disseminated multifocal Ewing sarcoma, identified several factors that significantly correlate with a worse outcome: patient age of more than 14 years at diagnosis, initial tumor volume of ≥200 mL, bone marrow metastases, and additional lung metastases [18,55]. Adverse clinical prognostic factors for patients with non-metastatic Ewing sarcoma are the site of the primary tumor (with axially located tumors with a poorer prognosis), poor histological response after neoadjuvant (radio-)chemotherapy (10% or greater viable tumor cells), and elevated serum lactate dehydrogenase levels [55,56,57]. Besides patient characteristics, the success of the local tumor therapy affects the global outcome [55,58]. About 26% of primary tumors are located in the pelvis [57,59]. Pelvic primary tumor manifestations have the least favorable prognosis compared with all other sites. They tend to relapse sooner, and they have a higher rate of local recurrence and lower disease-free and overall survival rates [60]: Guder et al. retrospectively analyzed 104 patients who had undergone tumor resection for pelvic Ewing sarcoma from 1988 to 2014. Surgical margins were free of tumor in 94.2%. Despite that, radiation therapy had additionally been performed in 77.9% of those patients. The response to chemotherapy was good in 78.8%. Local recurrence was detected in 7.7% of patients in this cohort. The most important negative predictor for overall survival was the presence of distant metastases at the time of surgery: while 5- and 10-year survival rates were 82.7% and 80.1% in non-metastasized pelvic Ewing sarcoma, these rates were only 61.4% and 41.6% in metastasized patients. Interestingly, patients with only a single distant metastasis at operation had a 5-year survival of 64.3% and a 10-year survival of 50.7% compared to only 50.0% and 16.7% in patients with multiple metastatic sites. While fair results appear thus to be achieved by a multimodal approach in non-metastasized Ewing sarcoma patients of the pelvis, in metastatic patients, the significance of complete tumor resection as part of the local treatment remains less certain. Guder et al. suggest that improved outcomes of combined local treatment approaches need to be weighed against these patients’ prognosis and quality of life [60].

### 3.2. Therapy

The total duration of the treatment is about 10 to 12 months. Neoadjuvant chemotherapy with vincristine, ifosfamide, doxorubicin, and etoposide (VIDE induction therapy) is administered in six chemotherapy cycles. This is followed by local therapy with complete surgical resection. If this is not possible, a combination of surgery and radiotherapy (45–54 Gray) is preferred to radiotherapy alone (60 Gray). Metastases present at the time of diagnosis are treated locally, which means surgically removed and/or irradiated. The adjuvant therapy comprises a combination of different cytostatics, which are repeatedly given, similar to the first chemotherapy phase. Eight chemotherapy blocks are currently standard. Common combinations of cytostatics are vincristine, actinomycin D, and cyclophosphamide (VAC) or vincristine, actinomycin D, and ifosfamide (VAI). Recent analyses have also highlighted that in Ewing sarcoma patients with pulmonary or pleural metastases, there is no clear benefit from busulfan-melphalan high-dose chemotherapy with autologous stem-cell rescue without whole-lung radiation [17]. Presently, there are new protocols for further international, randomized, controlled trials for the treatment of newly diagnosed EWING sarcoma, such as the EURO EWING 2012 [61].

#### Surgical Therapy

1. *Biological Reconstruction with Vascularized Grafts*

**Figure 3:**
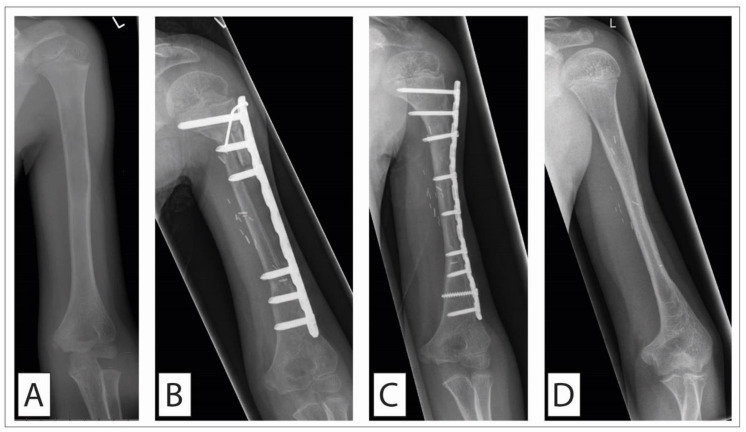
Ewing sarcoma of the humerus treated with a vascularized fibula graft. A 6-year old boy presented in the ward with persisting pain for 2 months in the left upper arm accompanied by intermittent fever. The plain a-p radiograph shows the central osteolysis within the diaphysis (**A**). Open biopsy was performed, showing Ewing sarcoma. After neoadjuvant chemotherapy, we performed complete en bloc resection (R0). The defect was reconstructed using a vascularized fibula graft by our colleagues from the department of plastic surgery (**B**). Temporary stabilization was performed with an LC-DCP plating. Seven months postoperatively, the fibula appeared radiologically fully osseointegrated distally, but a non-union with fracture of the plate was present proximally between the second and third screw. The LC-DCP was removed, and a titan elastic nail inserted. Postoperative care was a total cast of the left arm. Three months postoperatively, autologous bone from the iliac crest was placed at the non-union site followed by again plating of the humerus (**C**). (**D**) One year thereafter, the osteosynthesis material could be completely removed. The patient could freely use his arm, with, in the plain a-p radiograph, only a slimmer silhouette of the diaphysis still reminding of the performed procedure. Even when using a vascularized bone, as in the present case, bone healing can be challenging due to the immediate continuation of chemotherapy after wound healing.

In the context of limb-salvage surgery, reconstruction of bone defects with vascularized grafts, especially the fibula [62,63], plays an important role (an example is displayed in Figure 3). Gorski et al. [64] reported a case series of 25 free vascularized bone grafts (17 fibulae, 5 iliac crests, 3 medial femoral condyles). Seven of these patients had been treated for Ewing sarcoma. The rate of healing of the fibula grafts after a median of 5 months was 86%. In the total cohort, significant hypertrophy was observed in 13 patients. In a recent systematic review regarding free vascularized fibula grafting in the operative treatment of malignant bone tumors of the upper extremity, 56 studies were included [65]. The most frequent diagnosis at that site was osteosarcoma (35.1%), followed by giant cell tumor (25.3%), chondrosarcoma (17.7%), and Ewing sarcoma (11.1%). Most fibula grafts were placed in the humerus (57.3%). The median union rate of the free vascularized fibula grafts was 93.3% (median time to union, 5.0 months). Frequent complications were fracture (11.7%), nerve injury (7.5%), infection (5.7%), and hammer toe deformity (3.3%). Revision was necessary in 34.5% of all cases. The most commonly used assessment tool was the Musculoskeletal Tumor Society score, for which patients presented a median of 80% postoperatively. There was no significant difference in values for the Musculoskeletal Tumor Society score and the rates of union between patients who had received chemotherapy compared with those who did not receive chemotherapy.

For malignant tumors of the proximal humerus, as a local vascularized graft, the clavicula pro humero technique can be performed [66,67]. For flat bone defects such as in the skull or the scapula, the vascularized iliac bone can be used [68,69]. If necessary, these techniques can also be combined with free flap transfers [70].

2. *Biological Reconstruction with Allografts and Capanna Technique*

In the lower extremity, a single vascularized fibula graft does not meet weight-load requirements. Therefore, allograft reconstruction alone or combined with a vascularized fibula graft (Capanna technique) is a frequently described biological reconstruction technique [71]. In a recent systematic review on 25 articles describing this technique, the allograft with the vascularized fibula graft group had considerably lower rates of non-union (13%) in comparison to the allograft alone group (21.4%). Rates of infection (7.9% vs. 9%) and fracture (19.6% vs. 19.1%) were not significantly different. The allograft with vascularized fibula graft group also had significantly lower rates of explantation (6.57%) compared to the allograft-alone cohort (18.11%). Functional outcomes were similar across groups as measured by the Musculoskeletal Tumor Society score (88.22% vs. 87.77%) [72].

3. *Pelvic Ring Reconstruction Using Double-Barreled Free Vascularized Fibula Graft (FVFG)*

Reconstruction of the pelvic ring after resection of bone tumors is challenging. Erol et al. reviewed 16 children with pelvic Ewing sarcoma who had undergone pelvic ring reconstruction using double-barreled free vascularized fibula graft after sacroiliac resection. The fibula graft was placed between the supraacetabular region distally and the remaining ilium or sacrum proximally. The stability of the remaining pelvis and spinal column was secured by spinopelvic instrumentation. At the time of the final follow-up (mean, 49.8 months), 14 patients were alive while 2 patients had succumbed to the disease. The median Musculoskeletal Tumor Society score at that final follow-up was 80. The mean time for bone union was 9 months. Graft hypertrophy was found in all patients at 12 months. Seven patients had complications, three of whom required surgical revision due to one deep infection, one hematoma, and one wound dehiscence [73].

## 4. Chondrosarcoma

### 4.1. Prognosis

Chondrosarcomas are a heterogeneous group of malignant bone tumors that are characterized by the chondrocyte-derived production of a hyaline-like cartilage matrix [74]. They are often low- or intermediate-grade malignant tumors (Table 2) [75]. With a proportion of 20–27%, chondrosarcomas represent the second largest group of malignant bone neoplasms [1,2]. In this context, conventional chondrosarcoma represents the largest subgroup, representing 85% of chondrosarcomas [76]. Since 2002, low-grade chondrosarcomas (G1) are classified as locally aggressive and, as such, intermediate lesions (Table 3). These intermediate cartilaginous tumors should be termed atypical cartilaginous tumors in long and short tubular bones and “C1” in the axial skeleton [4,77]. As these are slow growing, after incomplete resection, 20–30% of the local relapses are only diagnosed after 5–10 years [78,79]. For this reason, an attentive and long follow-up needs to be performed [78]. Chondrosarcomas are typically localized in long bones, pelvic bones, and the scapula [80]. Grade 1 tumors often have mutations in the *SDH* gene family (central atypical cartilaginous tumors mostly *IDH1* and *2*, secondary peripheral atypical cartilaginous tumors mostly *EXT1*). Immunohistochemical stainings could show that chondrosarcomas are *S100* positive as well as negative for brachyury. In 86% of the cases, G2 and 3 tumors exhibit alterations of the *RB1* pathway. Due to the complexity and number of the described mutations, we refer to the revised WHO classification system for bone tumors of 2020 for further detail. Dedifferentiated chondrosarcoma is an exception here: it develops in 10–15% of cases of a central chondrosarcoma and it combines the histological criteria of a conventional chondrosarcoma and a high-grade noncartilaginous sarcoma [3].

Achieving negative margins is generally a challenge in the surgical treatment of malignancies of the axial skeleton and pelvis. Independent prognostic factors are tumor localization, tumor extent, age, and grading. Interestingly, it is still controversially discussed if local recurrence and distant metastasis represent independent prognostic factors [81,82,83,84]. However, local relapse represents a risk factor for metastasis [85]. Moreover, in central conventional chondrosarcoma, an axial location and a soft-tissue component of ≥1 cm represent strong risk factors for metastasis and mortality [86]. Given that approximately 50% of local relapses are asymptomatic, strict implementation of routine follow-up recommendations is strongly recommended [87]:

- G1/2: every 6 months for the first 2 years and then annually for another 10 years.
- G3 and dedifferentiated tumors: every 3 months for 3 years, every 4 months until completion of the 5th year, then every 6 months until completion of the 10th year.

A rise in incidences has been observed in Western health care systems over the past 30 years. Causes for this observation may be an extended life expectancy and an increase in incidental findings due to an increase in medical imaging [82].

### 4.2. Therapy

Depending on the grading, curettage or wide resection represents the standard of treatment. Recent data suggest a role for high-dose, advanced radiation therapies in selected high-risk chondrosarcoma patients with inoperable tumors, surgically challenging locations, or unplanned positive margins using photons or protons [88,89]. The efficacy of adjuvant chemotherapy is uncertain but may be considered in the management of patients with dedifferentiated chondrosarcoma [90]. Slow disease progression (low fraction of dividing cells), expression of the multidrug resistance 1 gene [91], and high activity of anti-apoptotic pathways (e.g., *Bcl-2* family members) [92] are considered responsible for chemotherapy resistance, especially in the conventional type and the rare clear cell variant. However, even for the rare high-grade tumors, there is also currently no strong evidence for a beneficial effect of a chemotherapy.

#### 4.2.1. Systemic Treatment

Cytotoxic chemotherapies generally do not show efficacy in chondrosarcoma. Patients aged 41–65 years with dedifferentiated chondrosarcoma can be treated according to the EURO-B.O.S.S. protocol, but a survival benefit due to this adjuvant therapy remains controversial [93]. For malignancies that cannot be treated surgically, antiangiogenic substances (e.g., pazopanib) can be attempted as rescue therapy (off-label) [94]. If an *IDH1* mutation is present, IDH1 inhibitors (e.g., ivosidenib) may be considered. Overall, the evidence for these substances is low [95,96].

#### 4.2.2. Surgical Treatment

**Figure 4:**
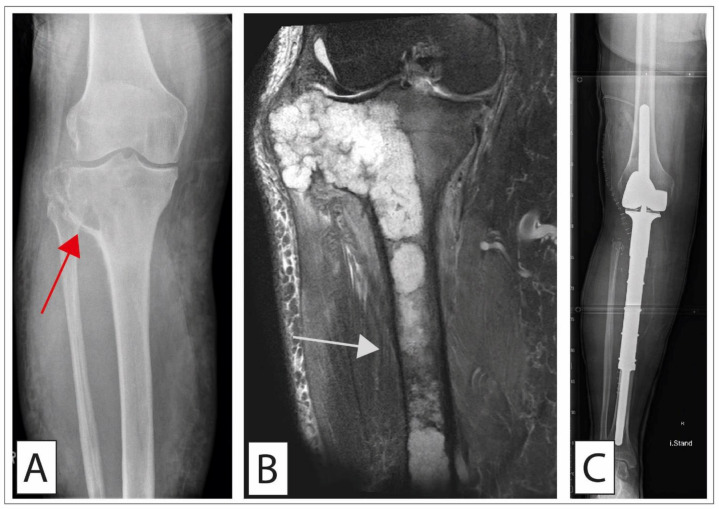
Chondrosarcoma treated with megaprosthesis. A 58-year-old patient presenting with a local prominence around the lateral knee. (**A**) Conventional radiographs showed an osteodestructive process in the region of the metaphysis affecting the bone cortex (red arrow). (**B**) A PDW-SPAIR MR coronal image illustrates the vast infiltration of the diaphysis distally (gray arrow). Open biopsy was performed, showing a highly differentiated chondrosarcoma (G1). Due to the destructive properties of this tumor, we performed complete resection (R0) and implanted a megaprosthesis matching the created defect (Implantcast, MUTARS proximal tibia) (**C**). A gastrocnemius and a soleus flap were performed to cover the foreign material at the site of the former tibia. The aponeurosis of the gastrocnemius was sutured on the patella tendon for additional stability of the extensor mechanism.

Grade 1 malignancies should be treated by an extensive intralesional curettage with local adjuvant chemical treatment or cryotherapy/cementation. Higher local relapse rates have been described for curettage compared to resection with free margins, which is why some authors advocate marginal resection in the trunk-near region. Dierselhuis et al., in a 2019 Cochrane review, failed to show advantages of wide resection over curettage for lesions of the long bones [97]. Grade 2–3 chondrosarcomas as well as local relapses with an M0 situation are treated by wide resection [87,98] (Figure 4). Overall, the location of the tumor (e.g., pelvis or joints) determines the complexity of the surgical treatment and may subsequently require sophisticated surgical reconstruction techniques. Interestingly, in a recent study, Song et al. demonstrated a survival benefit for complete resection of the primary tumor for conventional G2 chondrosarcoma including 200 patients with an M1 situation from the SEER database [99]. In such a case, the resection of the primary tumor can be discussed with the patient. Navigation-assisted surgery represents a new emerging surgical tool that may help to achieve clear margins especially in complex tumor situations such as in the pelvic region. Preliminary studies using this technique have already reported a trend towards improved local tumor control and longer tumor-free survival [100]. Another study, by Sambri et al., observed, in a small cohort of 61 patients with G2 or 3 chondrosarcomas, that pulmonary metastasectomy may be associated with prolonged survival, suggesting that pulmonary metastasis surgery in cases with isolated metastases may also be critically discussed with the patient [101].

#### 4.2.3. Surgical Treatment of Chondrosarcoma of the Pelvis

**Figure 5:**
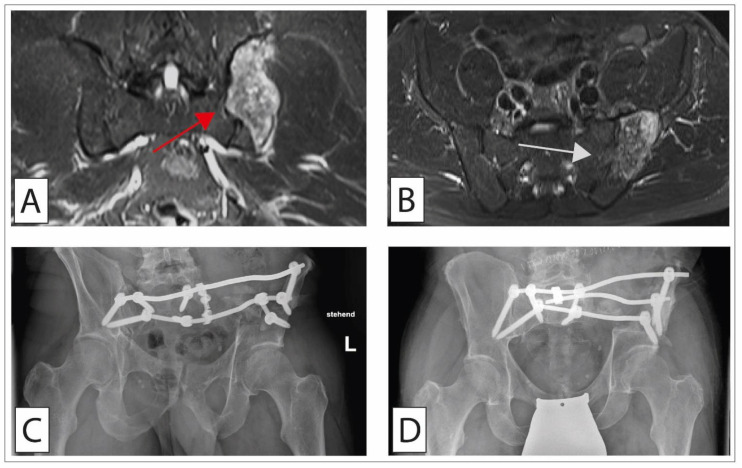
Chondrosarcoma of the pelvis treated with wide resection and reconstruction with allogenic and autologous bone graft. A 56-year-old patient presented in the ward with local low back pain. MR imaging showed a bone tumor in the postero-medial iliac crest (red arrow) with transgression of the sacroiliac joint (white arrow) (T2 tirm coronal (**A**) and axial (**B**)). Histopathological analyses after open biopsy showed a G1 chondrosarcoma with no other foci after staging. (**C**) En bloc resection was performed with a transiliacal osteotomy laterally and an osteotomy along the neuroforamina of the sacrum. Reconstruction was performed with a screw–rod system with three pedicle screws brought into the remaining iliac bone/acetabulum on the left and, on the right side, one S1 screw, one ilium screw, and one S2 ala-ilium screw. Allogenic bone was brought into the site of resection, augmented by an autologous rib. After 3 months of partial-weight bearing, the patient was allowed full-weight bearing. One year postoperatively, the patient had to be revised with a broken rod and incomplete osseointegration of the graft material. (**D**) Screw augmentation plus additional rod implantation were performed, accompanied by new autologous cancellous bone grafting. As an alternative procedure to the BMP-9 rich autologous rib, a vascularized fibula graft or the Capanna technique might have been used in this patient.

A unique situation is the presence of a central chondrosarcoma of the pelvic region (Figure 5). Chondrosarcomas of the pelvic region have an increased risk of metastasis and local recurrence (50% each). In addition, high demands are imposed on the surgeon for the surgical treatment since surgical procedures and reconstruction of the pelvic ring are highly complex [102]. A wide excision (>4 mm) should be the goal of surgical therapy, as smaller margins have an increased risk of early local relapse and of a reduced long-term survival [103]. At the same time, achieving these margins may entail the resection of nerves or nerve roots, which can be especially demanding for the patient in the case of the pudendal nerve. Subsequent reconstruction of the pelvic ring is challenging. Currently, reconstructions are performed by biological (e.g., hip transposition, massive allografts, autografts) or endoprosthetic reconstructions (e.g., allograft/prosthesis composites or prosthetic reconstructions). However, both allografts and autografts have a relevant risk of infection and/or fracture and often a postoperative leg length discrepancy is frequently observed [104]. Specially developed modular tumor prostheses demonstrated a reasonable functional outcome with long durability, with, however, also high infection rates [105]. Nevertheless, technical advances in design, structure, and fabrication have made such treatment strategies available for those patients affected by osteosarcoma of the pelvis.

#### 4.2.4. Radiotherapy

Traditionally, chondrosarcoma is considered to be little sensitive to radiation. However, Catanzano et al., in 2019, described, in a retrospective study analyzing 5427 patient data sets from the National Cancer Database (NCDB), that advanced radiotherapies such as intensity-modulated radiation therapy, proton-beam therapy, and stereotactic radiosurgery (with more than 60 Gy) could result in a survival benefit in high-risk chondrosarcoma patients (positive margins, central localization: spine, pelvis) [88]. As this was a retrospective study, evidence of a beneficial effect was not sufficiently provided; however, the authors recommend that an intensity-modulated radiation therapy should be discussed in cases of chondrosarcoma in the pelvis or axial skeleton and uncertain margin-free resection.

## 5. Current Trends and Future Perspectives

The successful treatment of hard-tissue sarcoma is still an interdisciplinary challenge. Despite substantial improvements in systemic chemotherapy and targeted radiation therapy, complete surgical resection of the tumor remains an integral part of most curative treatment regimens. The challenge to face the resection of these often already substantial tumor masses is two-fold: First, a complete R0 resection is necessary. This is then followed by a reconstruction of the created defect to minimize the resulting functional deficits. The more extensive the resection is, the more difficult also is the reconstruction. Keeping safety margins minimal without compromising the total resection of the tumor can help to keep also the required reconstructive efforts limited. Future possibilities may lie here in the field of augmented reality, where, after promising proof-of-concept trials, the first clinical studies are emerging (reviewed by [106]). Successful cadaveric studies using optically augmented reality, for example, showed improved resection results in bone tumors [107,108,109].

Another interesting new approach is to administer specific markers that enrich within the tumor or the healthy surrounding tissue, as was described for the distinction of viable from nonviable bone in the case of septic revision of hip arthroplasty using tetracycline as a fluorescent marker [110]. What can also now readily be used is the generation of 3D models to plan the intended procedure [111]. Especially for bone tumors, patient-specific resection guides based on preoperative imaging (patient-specific instrumentation) may possibly help to obtain precise and reliable resection results. Promising results have been reported for such an approach in pelvic bone tumors [112,113,114,115], a situation where clear margins are particularly difficult to achieve.

To reconstruct the generated bone defects, biological reconstruction and implantation of avital implants are the two main options a surgeon can choose from. While theoretically all conceivable defects could be filled with metal replacements, there are clear limitations to that approach: lacking proper muscle attachment and poor bone ingrowth with secondary aseptic loosening is still the harmless complication. Megaprostheses still keep being associated with high rates of infection. While these are estimated to be generally around 10% [116], even higher rates have been reported in the literature depending on the collective, surgical time, and surgical site [117,118], with the highest rates reported for pelvic implants, with infection rates of up to 42% [118]. For both complications, new design concepts offer a much better perspective for future patients treated with such implants: Patient-specific instrumentation and patient-specific implants can shorten the surgical time required for these procedures [119,120,121], thus also possibly reducing the risk of perioperative infection [118]. They also allow us to mechanically better address even complex defects than do prefabricated solutions, thus possibly also leading to better long-term survival. Crucial also for such long-term survival is excellent osseointegration of the implanted device. Osseointegration is thereby strongly influenced by the type of implant used. Especially β-titanium alloys such as Ti-6Al-4V offer excellent properties with respect to strength and corrosion resistance and they also possess a low elastic modulus favorable to the long-term integrity of the implant–bone interface [122]. The oxide layer formed on their surface due to their low electrical conductivity facilitates adhesion of osteoblasts when compared to other metals [123]. Further reduction in local fibrous tissue production and enhancement of cell adhesion and differentiation can be achieved by coating the implants, for example, with hydroxyapatite [124]. Creating favorable 3D-printed porous structures at the bone–implant interface can still lead to vastly improved osseointegration of implants [125]. Using scaffolds/3D structures at the bone–implant interface may also help to reduce the difference in elastic modulus and, as such, further prevent aseptic loosening [126]. In trauma surgery, for example, the use of an implant material isoelastic to bone (CFR-PEEK) could reduce the rate of secondary screw perforation after plating of proximal humerus fractures [127].

While anti-allergic surfaces consisting, for example, of Ti(Nb)N have been proposed for quite some time, especially in the field of primary arthroplasty [128], other techniques of surface modification are being discussed in the context of infection prevention [129,130]. Presently available on the market are silver and iodine coatings, gentamicin poly(D, L-lactide) (PLLA) coating, and a fast-resorbable hydrogel coating composed of covalently linked hyaluronan and PLLA [131]. In tumor surgery with implantation of megaprostheses, it has been reported that silver coating can reduce the risk of periprosthetic joint infection [132,133,134]. Additionally, a povidone-iodine coating of implants has, so far, showed promising results including tumor prostheses [135,136]. Recently, a new approach was suggested in which the implants are coated with a defensive antibacterial-coating hydrogel, which is supposed to reduce bacterial adhesion and biofilm formation [130]. Although the data on direct bacterial adhesion and biofilm formation on ceramic articulating surfaces in contrast to CoCr and Polyethylene remain controversial [137,138], the use of ceramic heads has been reported to be associated with a lower risk of revision for periprosthetic joint infection [139]. Hosman et al. [140] suggested that wear debris from metal-on-metal bearing surfaces might accelerate the growth of bacteria. Although, in the field of ceramics, this discussion of the role of the implant surface still remains open, it introduces an interesting perspective to prevent microbial infection: the generation of a microbially favorable nanosurface structure. First results in the literature suggest that superhydrophobic surfaces, also on implant materials such as titanium, could repel hydrophobic pathogens [141,142,143].

## 6. Conclusions

In bone sarcomas, successful surgical resection remains essential for long-term survival of the patients. Reconstruction of the created defects can be challenging and, sometimes, requires an interdisciplinary approach. Despite great advances over the past decades, both biological and artificial reconstruction strategies still have clear limitations with respect to function and long-term outcome. Current developments in surgical technique, material science, and implant design will still offer further improvements in the years to come.

## Figures and Tables

**Table 1:** The WHO classification of malignant chondrogenic and osteogenic as well as Ewing sarcomas [3,4].

Type	Subtype
Osteosarcoma	Low-grade central osteosarcoma
	Osteosarcoma NOS
	Conventional osteosarcoma
	Telangiectatic osteosarcoma
	Small cell osteosarcoma
	Parosteal osteosarcoma
	Periosteal osteosarcoma
	High-grade surface osteosarcoma
	Secondary osteosarcoma
Chondrogenic tumors	Chondrosarcoma, grade 1
	Chondrosarcoma, grade 2
	Chondrosarcomas, grade 3
	Periosteal chondrosarcoma
	Clear cell chondrosarcoma
	Mesenchymal chondrosarcoma
	Dedifferentiated chondrosarcoma
Undifferentiated small cell sarcomas	Ewing sarcoma (gene fusions involving genes of the FET family (e.g., EWSR1))

**Table 2:** Histopathological grading systems for bone tumors [3,9,11].

Category	WHO
Low-grade	Grade 1
High-grade	Grade 2
	Grade 3

**Table 3:** Prevalence, metastatic potential, and 10-year survival rate according to the tumor grading. (Abbreviation: ACT, atypical cartilaginous tumor).

Variable	ACT/G1	G2	G3/4
Proportion	90%		10%
Metastatic potential	<5%	<25%	up to 85%
10-year survival rate	83–95%	64–86%	26–55%
